# Physiological and pathological aspects of Aβ in iron homeostasis via 5'UTR in the APP mRNA and the therapeutic use of iron-chelators

**DOI:** 10.1186/1471-2202-9-S2-S2

**Published:** 2008-12-03

**Authors:** Yael Avramovich-Tirosh, Tamar Amit, Orit Bar-Am, Orly Weinreb, Moussa BH Youdim

**Affiliations:** 1 Department of Pharmacology, Eve Topf and USA NPF Centers of Excellence, Technion-Faculty of Medicine, 31096 Haifa, Israel

## Abstract

Many studies have highlighted the pathological involvement of iron accumulation and iron-related oxidative stress (OS) in Alzheimer's disease (AD). Iron was further demonstrated to modulate expression of the Alzheimer's amyloid precursor holo-protein (APP) by a mechanism similar to that of regulation of ferritin-L and -H mRNA translation through an iron-responsive element (IRE) in their 5' untranslated regions (UTRs). Here, we discuss two aspects of the link between iron and AD, in relation to the recently discovered IRE in the 5'UTR of APP mRNA. The first is the physiological aspect: a compensatory neuroprotective response of amyloid-β protein (Aβ) in reducing iron-induced neurotoxicity. Thus, given that Aβ possesses iron chelation sites, it is hypothesized that OS-induced intracellular iron may stimulate APP holo-protein translation (via the APP 5'UTR) and subsequently the generation of its cleavage product, Aβ, as a compensatory response that eventually reduces OS. The second is the pathological aspect: iron chelating compounds target the APP 5'UTR and possess the capacity to reduce APP translation, and subsequently Aβ levels, and thus represent molecules with high potential in the development of drugs for the treatment of AD.

## Introduction

There is increasing evidence that iron accumulation in the brain can cause a vast range of disorders of the central nervous system. It has become apparent that iron progressively accumulates in the brain with age [1,2], and that iron-induced oxidative stress (OS) can cause neurodegeneration [3]. Free iron induces OS through its interaction with hydrogen peroxide (Fenton reaction), resulting in increased formation of hydroxyl free radicals. Free radical-related OS causes molecular damage that can then lead to a critical failure of biological functions and ultimately cell death [4,5].

In Alzheimer's disease (AD) pathology, iron is significantly concentrated in and around amyloid senile plaques, and neurofibrillary tangles (NFTs), leading to alterations in the pattern of the interaction between iron regulatory proteins and their iron responsive elements (IREs), and disruption in the sequestration and storage of iron [6,7]. Also, high levels of iron have been reported in the amyloid plaques of the Tg2576 mouse model for AD, resembling those seen in the brains of AD patients [8]. In addition to the accumulation of iron in senile plaques, it was demonstrated that the amount of iron present in the AD neuropil is twice that found in the neuropil of non-demented brains [6]. Further studies have suggested that accumulated iron supports the AD pathology as a possible source of OS-dependent reactive oxygen radicals, demonstrating that neurons in AD brains experience high oxidative load [9-12]. *Post mortem *analysis of AD patients' brains have revealed activation of two enzymatic indicators of cellular OS: heme oxygenase-1 [13] and NADPH oxidase [14]. Also, heme oxygenase-1 was greatly enhanced in neurons and astrocytes of the hippocampus and cerebral cortex of AD subjects, co-localizing to senile plaques and NFTs [15]. A recent study reported that ribosomal RNA provided a binding site for redox-active iron and serves as a redox center within the cytoplasm of vulnerable neurons in AD brain, in advance of the appearance of morphological change indicating neurodegeneration [16]. In addition, other evidence suggests that the metabolism of iron is disrupted in AD. For example, the location of the iron-transport protein transferrin in senile plaques, instead of its regular location in the cytosol of oligodendrocytes, indicated that it becomes trapped within plaques while transporting iron between cells [17]. The mediator of iron uptake by cells, melanotransferrin, and the iron-storage protein ferritin are altered in AD and are expressed within reactive microglial cells that are present both in and around senile plaques [18,19].

Previous studies assessing the effects of certain genes encoding proteins involved in iron metabolism, such as hemochromatosis (*HFE*) and Transferrin (*TF*) genes, on the onset of AD have been contradictory [20,21]. At the biochemical level, iron was demonstrated to facilitate the aggregation of β-amyloid peptide (Aβ) and increase its toxicity [22]. Indeed, the iron chelator deferrioxamine (DFO) prevented the formation of β-pleated sheets of Aβ_1–42 _and dissolved preformed β-pleated sheets of plaque-like amyloid [23]. Also, iron induced aggregation of hyperphosphorylated τ (tau), the major constituent of NFTs [24].

A direct link between iron metabolism and AD pathogenesis was provided recently by Rogers *et al. *[25], who described the presence of an IRE in the 5' untranslated region (5'UTR) of the amyloid precursor protein (APP) transcript. Thus, APP 5'UTR is selectively responsive to intracellular iron levels in a pattern that reflects iron-dependent regulation of intracellular APP synthesis. Indeed, iron levels were shown to regulate translation of APP holo-protein mRNA in astrocytes [26] and neuroblastoma cells [25] by a mechanism similar to iron control of the translation of ferritin-L and -H mRNAs via IREs in their 5'UTRs.

**Figure 1:**
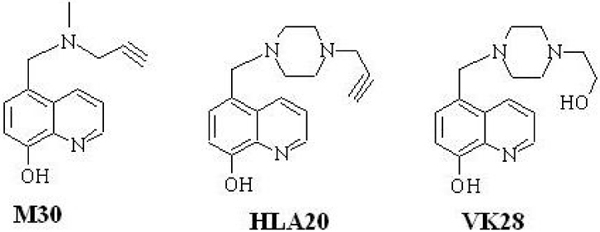
Chemical structures of the novel multifunctional iron chelators M30, HLA20 and VK28.

This review will discuss two main aspects of the link between iron and AD in relation to the recently discovered IRE in the 5'UTR of APP mRNA. First is the physiological aspect, which considers the neuroprotective response of Aβ in reducing iron-induced neurotoxicity. Thus, given that Aβ possesses iron chelating sites, it may be hypothesized that OS-induced intracellular iron levels stimulate APP holo-protein translation (via the APP 5'UTR) and the subsequent generation of its cleavage product, Aβ, as a compensatory response that eventually reduces OS. Second is the pathological aspect, which considers iron chelator compounds targeting the APP 5'UTR that possess the capacity to reduce APP translation, and subsequent Aβ generation, as molecules with high potential in the development of drugs for the treatment of AD (Figure 1).

## Aβ generation as a compensatory mechanism

Conflicting results have been reported in recent literature concerning whether the interaction between Aβ and iron is neurotoxic or neuroprotective [27]. Thus, while *in vitro *studies demonstrate that Aβ can induce OS and neurotoxicity (at high concentrations of aged Aβ), it is apparent from other reports that OS promotes Aβ generation (perhaps as a result of altered metal iron metabolism), presumably as a protective/compensatory response, leading to reduced neuronal OS. Consistent with this, amyloid plaques and NFTs in the cortex were found to be inversely correlated with OS markers (for example, 8-hydroxyguanosine) [28], indicating that oxidative damage is an early event in AD that decreases with disease progression. Indeed, in different APP transgenic mouse lines, the subtle functional deficits occur before the formation of amyloid plaques [29]. In addition, various sources of OS (for example, H_2_O_2_, UV, and reactive oxygen species (ROS)) have been demonstrated to increase neuronal Aβ production [30-32]. Moreover, Aβ has been shown to be upregulated by many forms of stress conditions, including apoptosis, ischemia, shortage of energy supply, hypoglycemia, and brain injury [33-35]. The levels of β-secretase were markedly increased by oxidative agents, with consequent augmentation of the levels of carboxy-terminal fragments of APP [36,37], further suggesting that OS may be the cause of Aβ production. Taken together, these studies suggest that Aβ formation may be a response to, rather than a cause of, neurotoxic oxidative challenge.

Previous studies have reported similarities between APP holo-protein and ferritin gene expression, both of which are driven by translational regulatory events [38]. The expression of the APP gene was up-regulated at the translational level by iron and interleukin-1, which was paralleled by the action of 5'UTR sequences that are similar to the 5'UTR sequences in the mRNA coding for the L- and H-subunits of ferritin. Evidently, IRE-dependent pathways govern the post-transcriptional expression of many proteins involved in iron metabolism, in addition to ferritin and transferrin receptor.

Aβ has been characterized as a metalloprotein that binds transition metal ions via three histidines and a tyrosine residue located in the hydrophilic amino-terminal part of the peptide. Thus, given that Aβ is a metalloprotein that possesses strong chelating properties for transition metal ions [27], it is proposed that Aβ generation under oxidative conditions may be aimed at sequestering metal ions in order to prevent further potentional oxidative damage. Consistent with this, previous studies demonstrated that the injection of Aβ-iron complexes into rat cerebral cortex was less toxic than iron alone [39]. Additionally, Aβ1–40 at 5 μM was found to protect primary neuronal cultures from the neurotoxicity of iron [40,41]. Recent findings demonstrated that three histidine residues in Aβ control the redox activity of iron, indicating that Aβ is likely to be an important antioxidant. Thus, it was shown that Fe^3+^-catalyzed ascorbate oxidation and hydroxyl radical generation were inhibited in the presence of Aβ1–40 or Aβ1–42 [42].

**Figure 2:**
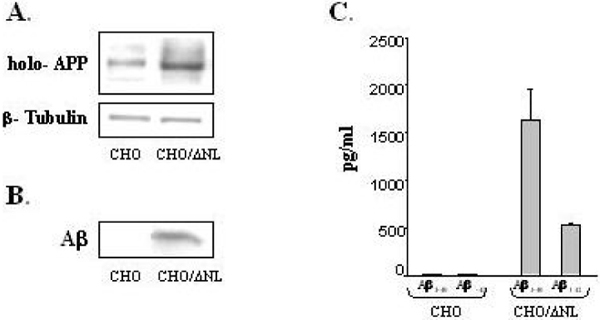
APP expression in CHO cells. **(A) **A representative western blot analysis of cellular holo-APP in CHO cells and CHO cells stably transfected with the APP 'Swedish' mutation (CHO/ΔNL). Cellular holo-APP was detected in cell lysates with 22C11 antibody (directed to the APP amino terminus). The loading of the lanes was normalized to levels of β-tubulin. **(B) **Aβ was detected in the medium of CHO and CHO/ΔNL cells by immunoprecipitation and western blotting with monoclonal antibody 6E10 (which recognizes an epitope within residues 1–17 of Aβ domain). **(C) **Aβ1–40 and Aβ1–42 levels were measured using standard sandwich ELISA (BioSource, Camarillo, California, USA).

**Figure 3:**
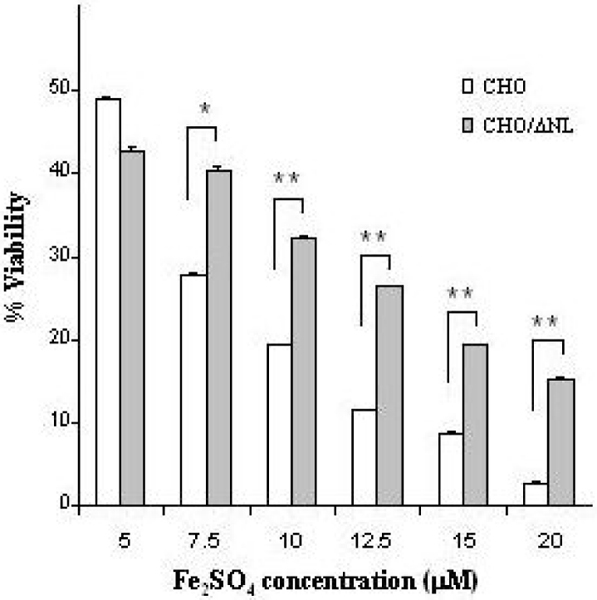
Comparison of viability between CHO and CHO/ΔNL cells following FeSO_4 _treatment. CHO and CHO/ΔNL cells were incubated in the absence (Control) or presence of different concentrations of FeSO_4 _(5–20 μM) for 24 h. Viability of the cell cultures was determined by the MTT (3-(4,5-dimethylthiazol-2-yl)-2,5-diphenyl tetrazolium bromide) method. The data are expressed as percent of Control. Representative curves from four independent experiments are shown. *P < 0.05; **P < 0.01 versus CHO cells.

**Figure 4:**
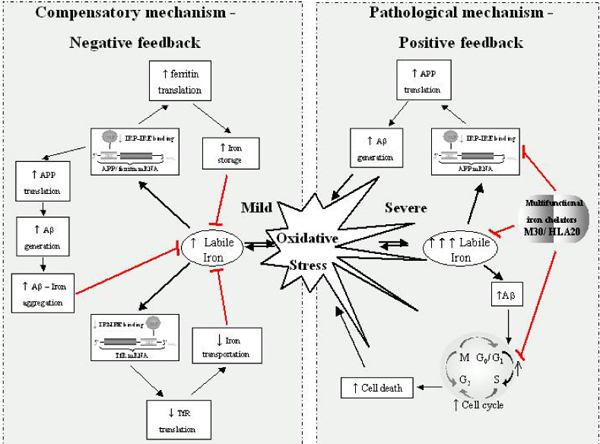
Schematic representation of physiological iron homeostasis mechanisms, including Aβ generation as a compensatory process that reduces OS damage and the pathological mechanisms of iron-induced neurodegeneration in AD and its prevention by iron chelators.

Here, we analyzed the capacity of Aβ to reduce iron-induced cell death in Chinese hamster ovary (CHO) cells stably transfected with the APP 'Swedish' mutation (CHO/ΔNL), which express elevated levels of holo-APP in cell lysate and Aβ1–40 and Aβ1–42 peptides in the medium compared to controls (Figure 2). Indeed, as demonstrated in Figure 3, CHO/ΔNL cells provided significant increased protection against iron-mediated cell toxicity compared to control CHO cells, further suggesting that Aβ significantly reduced the neurotoxicity of iron. Based on these findings, we hypothesize that the IRE of the 5'UTR of the APP transcript may be linked to the compensatory response of Aβ that helps neurons cope with altered iron homeostasis. Figure 4 illustrates the main physiological iron homeostatic mechanisms, including the IRE in the 5'UTR of the APP mRNA as a potential target involved in the compensatory response of Aβ. First, following moderate OS conditions and abnormal iron metabolism, APP synthesis is enhanced via the APP-5'UTR; second, as Aβ is a cleavage product of APP, the increase in APP level will be accompanied by elevation of Aβ generation; and third, Aβ peptides may have compensatory/neuroprotective properties as a result of their ability to trap free iron [27]. In addition to known iron regulation targets (for example, transferrin receptor, ferritin), the formation of Aβ peptides may be considered a novel compensatory response that reduces moderate OS damage.

## Iron chelation for the treatment of AD

Under extreme pathological conditions – that is, at some threshold level of ROS generation – it appears that the main role of Aβ switches from neuroprotective to dyshomeostatic in terms of cerebral biometals and APP/Aβ/metal-redox complexes, leading to a vicious cycle of increased ROS production and Aβ generation. Chelation has the potential to prevent iron-induced ROS, OS, and Aβ aggregation and, therefore, chelation therapy may be considered a valuable therapeutic strategy for AD. In fact, intramuscular administration of DFO, a potent iron chelator, slowed the clinical progression of AD dementia [43] and some success has also been achieved with clioquinol, another metal-complexion agent [44,45]. However, clioquinol is highly toxic and DFO has poor blood-brain barrier penetration.

The identification of an IRE in the 5'UTR of the APP transcript led to a novel therapeutic approach aimed at reducing amyloidosis by FDA pre-approved drugs targeted to the IRE in the APP mRNA 5'UTR [38]. For example, the APP 5'UTR-directed drugs DFO (Fe^3+ ^chelator), tetrathiomolybdate (Cu^2+ ^chelator), and dimercaptopropanol (Pb^2+ ^and Hg^2+ ^chelator) were found to suppress APP holo-protein expression and lower Aβ secretion [38,46,47]. In addition, the bi-functional molecule XH-1, which contains both amyloid-binding and metal-chelating moieties, was shown to reduce APP expression in SH-SY5Y cells and attenuate cerebral Aβ in PS1/APP transgenic mice [48]. Additional drug classes were also reported to suppress the APP 5'UTR and limit APP expression, including antibiotics, selective serotonin reuptake inhibitors, and other selective receptor antagonists and agonists [47].

The concept of metal chelators for clinical use in neurological disorders that could remove excess iron in the brain recently led our group to develop non-toxic, lipophilic, and brain permeable iron chelators for neurodegenerative diseases. The novel iron chelator VK28 (varinel) [49] and the multi-functional drugs HLA20 and M30 (Figure 1) [50], which possess the potent iron chelating activities and neuroprotective properties of N-propargylamine, were recently shown to induce a significant down-regulation of membrane-associated holo-APP levels in the mouse hippocampus and in human SH-SY5Y neuroblastoma cells, presumably by chelating intracellular iron pools [51]. Indeed, the iron chelator drugs VK28, HLA20, and M30 (Figure 1) were found to suppress translation of a luciferase reporter mRNA via the APP 5'UTR sequence (Table 1) [51]. Furthermore, M30 markedly reduced the levels of the amyloidogenic Aβ in the medium of CHO cells stably transfected with the APP 'Swedish' mutation (CHO/ΔNL) [51]. In addition, naturally occurring polyphenols – for example, EGCG ((-)-epigallocatechin-3-gallate)*a*nd curcumin – might be used as another novel and promising therapeutic approach for treating AD. Both compounds have well characterized antioxidant and metal-chelating (iron and copper) activities [52-54] and have been demonstrated to exert neuroprotective activity against a variety of neurotoxic insults, as well as to regulate APP processing and Aβ burden in cell culture and *in vivo *[55]. EGCG treatment led to a reduction of Aβ levels in CHO/ΔNL [56], murine neuron-like cells (N2a) transfected with the APP 'Swedish' mutation and primary neurons derived from 'Swedish' mutant APP-over-expressing mice [57]. *In vivo*, EGCG significantly reduced cerebral Aβ levels concomitant with reduced Aβ amyloid plaques in TgAPPsw transgenic mice overproducing Aβ [57].

**Table 1:** Effect of M30 and HLA20 on APP 5'UTR- conferred translation of a luciferase reporter mRNA

**Drug**	**Inhibition (% of control)**
M30 (20 μM)	7.1 ± 1.07 *
M30 (100 μM)	33.7 ± 8.3^†^
HLA20 (20 μM)	16.2 ± 2.4^†^
HLA20 (100 μM)	27.3 ± 2.9 *
VK28 (20 μM)	26.6 ± 7.7^†^
VK28 (100 μM)	40.2 ± 7.5^†^

Since there is a functional IRE in the 5'UTR of APP mRNA, we further investigated the efficacy of the iron chelator drugs M30, HLA20 and VK28 to modulate the translation of a luciferase reporter gene driven by APP 5'UTR sequences. The assay was performed essentially as described in Reznichenko *et al*. [56]. The pGALA construct was generously provided by JT Rogers (Massachusetts General Hospital, Boston, MA, USA). Human U-87-MG glioma cells, selectively chosen for their high transfection efficiency, were grown in flasks (100 mm^2^) and transfected with 7 μg of DNA from the parental vector pGL-3 or pGALA constructs and co-transfected with 3 μg of DNA from a construct that expresses green fluorescent protein (GFP) to standardize for transfection efficiency. The pGALA consists of a pGL-3 backbone to which the APP 5'UTR sequences (containing the IRE) were inserted in front of the luciferase gene start codon and the complete APP 3'UTR sequences immediately downstream of the luciferase, to provide the natural arrangement of the APP gene 5' and 3'. After 12 h, the cells were split equally into 96-well plates and grown without (control) or with the iron chelator drugs M30, HLA20 or VK28 for 48 h. Cell viability was established by a fluorescent microscopic examination of each well, quantified for GFP activity at 480/509 nm wavelength (using an automatic Wallac-1420 multilabel counter) and then lysed to determine luciferase activity. For statistical analysis one-way ANOVA followed by Student's *t*-test was performed. Quantitative values are mean ± standard error of the mean (n = 3). ^†^*P *< 0.05, *P < 0.01 versus control was considered significant.

Our recent studies have shown that prolonged administration of EGCG to mice induced a reduction in holo-APP levels in the hippocampus [58]. In SH-SY5Y cells, EGCG significantly reduced both the mature and full-length cellular holo-APP without altering APP mRNA levels, as shown by two-dimensional gel electrophoresis, suggesting a post-transcriptional action [56]. Indeed, we demonstrated that EGCG reduced the translation of a luciferase reporter gene fused to the APP mRNA 5'UTR, which includes the APP IRE [56,58]. The observation that the alteration in APP following treatment with EGCG was blocked by exogenous iron provides further support to the implication of the metal-chelating property of EGCG in the regulation of iron homeostasis-associated proteins. A recent study reported the development of a high-throughput screen (library of 110,000 compounds from the Laboratory for Drug Discovery on Neurodegeneration) to identify APP mRNA 5'UTR-directed compounds that may be developed into therapeutic agents for AD [59]. Table 2 summarizes the effects of various compounds (1–50 μM) that were demonstrated to limit APP 5'UTR-conferred translation on holo-APP and Aβ levels.

**Table 2:** Summary of the effect of various drugs/compounds targeted to the IRE in the APP mRNA 5'UTR on holo-APP and Aβ levels

Drug/compound	Mechanism of action	Holo-APP levels	Aβ levels	Reference
M30	Novel multifunctional iron chelator	↓	↓	[51]
VK28	Novel multifunctional iron chelator	↓	↓	Unpublished data
HLA20	Novel multifunctional iron chelator	↓	↓	Unpublished data
EGCG	Main polyphenol constituent of green tea	↓	↓	[56]
				
**FDA approved drugs**		↓	↓	
DFO	Prototype iron chelator	↓	↓	[25]
Paroxetine	SSRI and chelator	↓	↓	[47,65]
Dimercapropanol	Hg^2+ ^and Pb^2+ ^chelator	↓	↓	[47]
Phenserine	Anticholinesterase	↓	↓	[66]
Tetrathiomolybdate	Cu^2+ ^chelator	↓	↓	[38]
Azithromycin	Macrolide antibiotic	↓	↓	[38]
Erythromycin	Macrolide antibiotic	↓	↓	[65]
N-acetyl cysteine	Antioxidant	↓	↓	[65]
XH-1	Bi-functional metal chelator	↓	↓	[48]

Arrows indicate decreased levels. SSRI, selective serotonin reuptake inhibitor.

Finally, a new, additional aspect of iron chelator compounds in the etiology of AD therapy is related to their ability to abort anomalous cell cycle re-activation in post-mitotic degenerating neurons. Indeed, during the last few years accumulating evidence for an activated cell cycle in the vulnerable neuronal population in AD has suggested a crucial role for cell cycle abnormalities in AD pathogenesis. Therefore, therapeutic interventions targeted towards ameliorating mitotic changes would be predicted to have a positive impact on AD progression. Previous studies have shown that the re-activation of the cell cycle is an obligatory component of the apoptotic pathway evoked by Aβ [60-62]. Recently, we have found that M30 (0.1 μM) significantly reduced the percentage of neurons in S phase (approximately 50%), while increasing their relative cell number again in G_0_/G_1 _phase (approximately 1.4-fold) and lowered apoptotic levels after exposure to Aβ25–35 (25 μM) in primary cultures of rat cortical neurons. In support, the novel iron chelator drugs were previously shown to induce cell cycle arrest; M30 and HLA20 increased the number of PC12 cells in G_0_/G_1_, and decreased the cell number in S phase, as well as the proportion of cells in the G_2 _phase, further indicating that both compounds inhibited cell progress beyond the G_0_/G_1 _phase.

We recently presented a novel neuroprotective target for iron chelators with regard to the aberrant cell cycle reentry of postmitotic neurons in AD. Accordingly, similar to cancer drug therapy, a newly therapeutic strategy for neurodegenerative diseases is currently directed at interfering with mitogenic signaling and cell cycle progression to ameliorate cell death. Because iron chelators have been shown to affect critical regulatory molecules involved in cell cycle arrest and proliferation [55], a therapeutic intervention with these compounds is assumed to have a profound impact on neuron preservation and AD progression. Indeed, our studies revealed that the multi-functional iron chelators M30 and HLA20 [51], as well as EGCG [63], induced differentiation features in neuroblastoma and PC12 cells, including cell body elongation, stimulation of neurite outgrowth, and up-regulation of the growth associated protein-43 (GAP-43). Taken together, the data suggest that iron chelators may be considered potential therapeutic agents in AD, targeting early cell cycle anomalies, and re-establishing the lost synaptic connection in the injured neuronal cells.

## Conclusion

The presence of an IRE stem-loop in the APP transcript suggests that this ubiquitous membrane-associated protein, as well as its cleavage product, Aβ, may have a significant role in iron homeostasis, as already exemplified by other iron-associated proteins. Thus, regarding the physiological pathway, Aβ production may be considered a compensatory or neuroprotective response that reduces OS damage. In the pathological aspect, novel therapeutic strategies may comprise iron chelating agents targeted to the IRE in the 5'UTR of the APP mRNA, specifically preventing iron-induced toxicity and over-production of Aβ. We have recently designed and synthesized several novel antioxidant/iron chelators with an 8-hydroxyquinoline moiety, and demonstrated their capacity to lower the expression of APP and the generation of Aβ. These latest findings implicate the therapeutic potential of our drugs as iron-chelator candidates targeting the regulation of APP/Aβ in AD.

In addition, considering a recent report describing a putative IRE in the 5'UTR of Parkinson's disease related α-synuclein mRNA [64], a parallel can be drawn between APP and α-synuclein both in the physiological and pathological aspects with respect to iron regulation. Indeed, it can be predicted that this RNA structure may have the potential to function as a post-transcriptional regulator of α-synuclein protein synthesis by age-related iron and redox pathophysiology upstream of neurodegeneration.

## List of abbreviations used

Aβ: β-amyloid; AD: Alzheimer's disease; APP: amyloid precursor protein; CHO: Chinese hamster ovary; DFO: deferrioxamine; EGCG: (-)-epigallocatechin-3-gallate; IRE: iron responsive element; NFT: neurofibrillary tangle; OS: oxidative stress; ROS: reactive oxygen species; UTR: untranslated region.

## Competing interests

The authors declare that they have no competing interests.

## References

[B1] Bartzokis G, Mintz J, Sultzer D, Marx P, Herzberg JS, Phelan CK, Marder SR (1994). *In vivo *MR evaluation of age-related increases in brain iron. AJNR Am J Neuroradiol.

[B2] Bartzokis G, Beckson M, Hance DB, Marx P, Foster JA, Marder SR (1997). MR evaluation of age-related increase of brain iron in young adult and older normal males. Magn Reson Imaging.

[B3] Zecca L, Youdim MB, Riederer P, Connor JR, Crichton RR (2004). Iron, brain ageing and neurodegenerative disorders. Nat Rev Neurosci.

[B4] Halliwell B (2001). Role of free radicals in the neurodegenerative diseases: therapeutic implications for antioxidant treatment. Drugs Aging.

[B5] Sayre LM, Smith MA, Perry G (2001). Chemistry and biochemistry of oxidative stress in neurodegenerative disease. Curr Med Chem.

[B6] Lovell MA, Robertson JD, Teesdale WJ, Campbell JL, Markesbery WR (1998). Copper, iron and zinc in Alzheimer's disease senile plaques. J Neurol Sci.

[B7] Pinero DJ, Hu J, Connor JR (2000). Alterations in the interaction between iron regulatory proteins and their iron responsive element in normal and Alzheimer's diseased brains. Cell Mol Biol.

[B8] Smith MA, Hirai K, Hsiao K, Pappolla MA, Harris PL, Siedlak SL, Tabaton M, Perry G (1998). Amyloid-beta deposition in Alzheimer transgenic mice is associated with oxidative stress. J Neurochem.

[B9] Moreira PI, Siedlak SL, Aliev G, Zhu X, Cash AD, Smith MA, Perry G (2005). Oxidative stress mechanisms and potential therapeutics in Alzheimer disease. J Neural Transm.

[B10] Castellani RJ, Honda K, Zhu X, Cash AD, Nunomura A, Perry G, Smith MA (2004). Contribution of redox-active iron and copper to oxidative damage in Alzheimer disease. Ageing Res Rev.

[B11] Honda K, Casadesus G, Petersen RB, Perry G, Smith MA (2004). Oxidative stress and redox-active iron in Alzheimer's disease. Ann N Y Acad Sci.

[B12] Casadesus G, Smith MA, Zhu X, Aliev G, Cash AD, Honda K, Petersen RB, Perry G (2004). Alzheimer disease: evidence for a central pathogenic role of iron-mediated reactive oxygen species. J Alzheimers Dis.

[B13] Takeda A, Smith MA, Avila J, Nunomura A, Siedlak SL, Zhu X, Perry G, Sayre LM (2000). In Alzheimer's disease, heme oxygenase is coincident with Alz50, an epitope of tau induced by 4-hydroxy-2-nonenal modification. J Neurochem.

[B14] Shimohama S, Tanino H, Kawakami N, Okamura N, Kodama H, Yamaguchi T, Hayakawa T, Nunomura A, Chiba S, Perry G (2000). Activation of NADPH oxidase in Alzheimer's disease brains. Biochem Biophys Res Commun.

[B15] Schipper HM (2000). Heme oxygenase-1: role in brain aging and neurodegeneration. Exp Gerontol.

[B16] Honda K, Smith MA, Zhu X, Baus D, Merrick WC, Tartakoff AM, Hattier T, Harris PL, Siedlak SL, Fujioka H (2005). Ribosomal RNA in Alzheimer disease is oxidized by bound redox-active iron. J Biol Chem.

[B17] Connor JR, Menzies SL, St Martin SM, Mufson EJ (1992). A histochemical study of iron, transferrin, and ferritin in Alzheimer's diseased brains. J Neurosci Res.

[B18] Yamada T, Tsujioka Y, Taguchi J, Takahashi M, Tsuboi Y, Moroo I, Yang J, Jefferies WA (1999). Melanotransferrin is produced by senile plaque-associated reactive microglia in Alzheimer's disease. Brain Res.

[B19] Grundke-Iqbal I, Fleming J, Tung YC, Lassmann H, Iqbal K, Joshi JG (1990). Ferritin is a component of the neuritic (senile) plaque in Alzheimer dementia. Acta Neuropathol.

[B20] Moalem S, Percy ME, Andrews DF, Kruck TP, Wong S, Dalton AJ, Mehta P, Fedor B, Warren AC (2000). Are hereditary hemochromatosis mutations involved in Alzheimer disease?. Am J Med Genet.

[B21] Berlin D, Chong G, Chertkow H, Bergman H, Phillips NA, Schipper HM (2004). Evaluation of HFE (hemochromatosis) mutations as genetic modifiers in sporadic AD and MCI. Neurobiol Aging.

[B22] Bush AI (2003). The metallobiology of Alzheimer's disease. Trends Neurosci.

[B23] House E, Collingwood J, Khan A, Korchazkina O, Berthon G, Exley C (2004). Aluminium, iron, zinc and copper influence the in vitro formation of amyloid fibrils of Abeta42 in a manner which may have consequences for metal chelation therapy in Alzheimer's disease. J Alzheimers Dis.

[B24] Yamamoto A, Shin RW, Hasegawa K, Naiki H, Sato H, Yoshimasu F, Kitamoto T (2002). Iron (III) induces aggregation of hyperphosphorylated tau and its reduction to iron (II) reverses the aggregation: implications in the formation of neurofibrillary tangles of Alzheimer's disease. J Neurochem.

[B25] Rogers JT, Randall JD, Cahill CM, Eder PS, Huang X, Gunshin H, Leiter L, McPhee J, Sarang SS, Utsuki T (2002). An iron-responsive element type II in the 5'-untranslated region of the Alzheimer's amyloid precursor protein transcript. J Biol Chem.

[B26] Rogers JT, Leiter LM, McPhee J, Cahill CM, Zhan SS, Potter H, Nilsson LN (1999). Translation of the alzheimer amyloid precursor protein mRNA is up-regulated by interleukin-1 through 5'-untranslated region sequences. J Biol Chem.

[B27] Atwood CS, Obrenovich ME, Liu T, Chan H, Perry G, Smith MA, Martins RN (2003). Amyloid-beta: a chameleon walking in two worlds: a review of the trophic and toxic properties of amyloid-beta. Brain Res Brain Res Rev.

[B28] Nunomura A, Perry G, Aliev G, Hirai K, Takeda A, Balraj EK, Jones PK, Ghanbari H, Wataya T, Shimohama S (2001). Oxidative damage is the earliest event in Alzheimer disease. J Neuropathol Exp Neurol.

[B29] Moechars D, Dewachter I, Lorent K, Reverse D, Baekelandt V, Naidu A, Tesseur I, Spittaels K, Haute CV, Checler F (1999). Early phenotypic changes in transgenic mice that overexpress different mutants of amyloid precursor protein in brain. J Biol Chem.

[B30] Misonou H, Morishima-Kawashima M, Ihara Y (2000). Oxidative stress induces intracellular accumulation of amyloid beta-protein (Abeta) in human neuroblastoma cells. Biochemistry.

[B31] Paola D, Domenicotti C, Nitti M, Vitali A, Borghi R, Cottalasso D, Zaccheo D, Odetti P, Strocchi P, Marinari UM (2000). Oxidative stress induces increase in intracellular amyloid beta-protein production and selective activation of betaI and betaII PKCs in NT2 cells. Biochem Biophys Res Commun.

[B32] Zhang L, Zhao B, Yew DT, Kusiak JW, Roth GS (1997). Processing of Alzheimer's amyloid precursor protein during H2O2-induced apoptosis in human neuronal cells. Biochem Biophys Res Commun.

[B33] Abe K, St George-Hyslop PH, Tanzi RE, Kogure K (1991). Induction of amyloid precursor protein mRNA after heat shock in cultured human lymphoblastoid cells. Neurosci Lett.

[B34] Shi J, Xiang Y, Simpkins JW (1997). Hypoglycemia enhances the expression of mRNA encoding beta-amyloid precursor protein in rat primary cortical astroglial cells. Brain Res.

[B35] Shi J, Perry G, Smith MA, Friedland RP (2000). Vascular abnormalities: the insidious pathogenesis of Alzheimer's disease. Neurobiol Aging.

[B36] Tamagno E, Bardini P, Obbili A, Vitali A, Borghi R, Zaccheo D, Pronzato MA, Danni O, Smith MA, Perry G (2002). Oxidative stress increases expression and activity of BACE in NT2 neurons. Neurobiol Dis.

[B37] Tamagno E, Parola M, Bardini P, Piccini A, Borghi R, Guglielmotto M, Santoro G, Davit A, Danni O, Smith MA (2005). Beta-site APP cleaving enzyme up-regulation induced by 4-hydroxynonenal is mediated by stress-activated protein kinases pathways. J Neurochem.

[B38] Rogers JT, Lahiri DK (2004). Metal and inflammatory targets for Alzheimer's disease. Curr Drug Targets.

[B39] Robinson SR, Bishop GM (2002). Abeta as a bioflocculant: implication for the amyloid hypothesis of Alzheimer's disease. Neurobiol Aging.

[B40] Zou K, Gong JS, Yanagisawa K, Michikawa M (2002). A novel function of monomeric amyloid beta-protein serving as an antioxidant molecule against metal-induced oxidative damage. J Neurosci.

[B41] Bishop GM, Robinson SR (2004). The amyloid paradox: amyloid-beta-metal complexes can be neurotoxic and neuroprotective. Brain Pathol.

[B42] Nakamura M, Shishido N, Nunomura A, Smith MA, Perry G, Hayashi Y, Nakayama K, Hayashi T (2007). Three histidine residues of amyloid-beta peptide control the redox activity of copper and iron. Biochemistry.

[B43] Crapper McLachlan DR, Dalton AJ, Kruck TP, Bell MY, Smith WL, Kalow W, Andrews DF (1991). Intramuscular desferrioxamine in patients with Alzheimer's disease. Lancet.

[B44] Cherny RA, Atwood CS, Xilinas ME, Gray DN, Jones WD, McLean CA, Barnham KJ, Volitakis I, Fraser FW, Kim Y (2001). Treatment with a copper-zinc chelator markedly and rapidly inhibits beta-amyloid accumulation in Alzheimer's disease transgenic mice. Neuron.

[B45] Ritchie CW, Bush AI, Mackinnon A, Macfarlane S, Mastwyk M, MacGregor L, Kiers L, Cherny R, Li QX, Tammer A (2003). Metal-protein attenuation with iodochlorhydroxyquin (clioquinol) targeting Abeta amyloid deposition and toxicity in Alzheimer disease: a pilot phase 2 clinical trial. Arch Neurol.

[B46] Rogers JT, Randall JD, Eder PS, Huang X, Bush AI, Tanzi RE, Venti A, Payton SM, Giordano T, Nagano S (2002). Alzheimer's disease drug discovery targeted to the APP mRNA 5'untranslated region. J Mol Neurosci.

[B47] Payton S, Cahill CM, Randall JD, Gullans SR, Rogers JT (2003). Drug discovery targeted to the Alzheimer's APP mRNA 5'-untranslated region: the action of paroxetine and dimercaptopropanol. J Mol Neurosci.

[B48] Dedeoglu A, Cormier K, Payton S, Tseitlin KA, Kremsky JN, Lai L, Li X, Moir RD, Tanzi RE, Bush AI (2004). Preliminary studies of a novel bifunctional metal chelator targeting Alzheimer's amyloidogenesis. Exp Gerontol.

[B49] Ben-Shachar D, Kahana N, Kampel V, Warshawsky A, Youdim MBH (2004). Neuroprotection by a novel brain permeable iron chelator, VK-28, against 6-hydroxydopamine lession in rats. Neuropharmacology.

[B50] Zheng H, Weiner LM, Bar-Am O, Epsztejn S, Cabantchik ZI, Warshawsky A, Youdim MB, Fridkin M (2005). Design, synthesis, and evaluation of novel bifunctional iron-chelators as potential agents for neuroprotection in Alzheimer's, Parkinson's, and other neurodegenerative diseases. Bioorg Med Chem.

[B51] Avramovich-Tirosh Y, Amit T, Bar-Am O, Zheng H, Fridkin M, Youdim MBH (2007). Therapeutic targets and potential of the novel brain-permeable multifunctional iron chelator-monoamine oxidase inhibitor drug, M-30, for the treatment of Alzheimer's disease. J Neurochem.

[B52] Guo Q, Zhao B, Li M, Shen S, Xin W (1996). Studies on protective mechanisms of four components of green tea polyphenols against lipid peroxidation in synaptosomes. Biochimica et Biophysica Acta.

[B53] Kumamoto M, Sonda T, Nagayama K, Tabata M (2001). Effects of pH and metal ions on antioxidative activities of catechins. Biosci Biotechnol Biochem.

[B54] Baum L, Ng A (2004). Curcumin interaction with copper and iron suggests one possible mechanism of action in Alzheimer's disease animal models. J Alzheimers Dis.

[B55] Mandel S, Weinreb O, Reznichenko L, Kalfon L, Amit T (2006). Green tea catechins as brain-permeable, non toxic iron chelators to 'iron out iron' from the brain. J Neural Transm.

[B56] Reznichenko L, Amit T, Zheng H, Avramovich-Tirosh Y, Youdim MB, Weinreb O, Mandel S (2006). Reduction of iron-regulated amyloid precursor protein and beta-amyloid peptide by (-)-epigallocatechin-3-gallate in cell cultures: implications for iron chelation in Alzheimer's disease. J Neurochem.

[B57] Rezai-Zadeh K, Shytle D, Sun N, Mori T, Hou H, Jeanniton D, Ehrhart J, Townsend K, Zeng J, Morgan D (2005). Green tea epigallocatechin-3-gallate (EGCG) modulates amyloid precursor protein cleavage and reduces cerebral amyloidosis in Alzheimer transgenic mice. J Neurosci.

[B58] Avramovich-Tirosh Y, Reznichenko L, Amit T, Zheng H, Fridkin M, Mandel S, Youdim MBH (2007). Neurorescue activity, APP regulation and amyloid- peptide reduction by novel multi-functional brain permeable iron- chelator- antioxidants, M-30 and green tea polyphenol, EGCG. Curr Alzheimer Res.

[B59] Bandyopadhyay S, Ni J, Ruggiero A, Walshe K, Rogers MS, Chattopadhyay N, Glicksman MA, Rogers JT (2006). A high-throughput drug screen targeted to the 5'untranslated region of Alzheimer amyloid precursor protein mRNA. J Biomol Screen.

[B60] Copani A, Condorelli F, Caruso A, Vancheri C, Sala A, Giuffrida Stella AM, Canonico PL, Nicoletti F, Sortino MA (1999). Mitotic signaling by beta-amyloid causes neuronal death. FASEB J.

[B61] Giovanni A, Wirtz-Brugger F, Keramaris E, Slack R, Park DS (1999). Involvement of cell cycle elements, cyclin-dependent kinases, pRb, and E2F × DP, in B-amyloid-induced neuronal death. J Biol Chem.

[B62] Wu Q, Combs C, Cannady SB, Geldmacher DS, Herrup K (2000). Beta-amyloid activated microglia induce cell cycling and cell death in cultured cortical neurons. Neurobiol Aging.

[B63] Reznichenko L, Amit T, Youdim MB, Mandel S (2005). Green tea polyphenol (-)-epigallocatechin-3-gallate induces neurorescue of long-term serum-deprived PC12 cells and promotes neurite outgrowth. J Neurochem.

[B64] Friedlich AL, Tanzi RE, Rogers JT (2007). The 5'-untranslated region of Parkinson's disease alpha-synuclein messenger RNA contains a predicted iron responsive element. Mol Psychiatry.

[B65] Tucker S, Ahl M, Bush A, Westaway D, Huang X, Rogers JT (2005). Pilot study of the reducing effect on amyloidosis *in vivo *by three FDA pre-approved drugs via the Alzheimer's APP 5' untranslated region. Curr Alzheimer Res.

[B66] Shaw KT, Utsuki T, Rogers J, Yu QS, Sambamurti K, Brossi A, Ge YW, Lahiri DK, Greig NH (2001). Phenserine regulates translation of beta-amyloid precursor protein mRNA by a putative interleukin-1 responsive element, a target for drug development. Proc Natl Acad Sci USA.

